# Conformational Dynamics of Plasmepsin X during Inhibitor Binding

**DOI:** 10.1021/acs.jpcb.6c00091

**Published:** 2026-05-13

**Authors:** Wilson Karubiu, Richard Kullmann, Michael Krummhaar, Christian Roth, Thomas R. Weikl

**Affiliations:** 28321 Max Planck Institute of Colloids and Interfaces, Department of Biomolecular Systems, Am Mühlenberg 1, Potsdam 14476, Germany

## Abstract

The aspartic protease plasmepsin X (PMX) of the parasite *Plasmodium* is a promising drug target for novel malaria therapies. Two potent inhibitors of PMX are WM382 and WM4, which both include a guanidinium group that is in contact with the two catalytic aspartates of PMX in the bound complexes. In structural representations of the inhibitors, the guanidinium group is typically depicted as uncharged. However, p*K*_*a*_ predictions with standard tools presented in this article indicate that the guanidinium groups of WM382 and WM4 are protonated and, thus, positively charged in the bound complexes. This positive charge is counterbalanced by a negatively charged catalytic aspartate D266 in PMX of *Plasmodium falciparum*, while the second catalytic aspartate D457 is uncharged. To investigate the interplay of the conformational dynamics of PMX and inhibitor (un)­binding, we performed Hamiltonian replica exchange molecular dynamics (H-REMD) simulations starting from the predicted protonation state of the PMX-WM382 complex. On eight unbinding pathways enabled by weakened interactions of PMX and WM382 in the H-REMD simulations, we observed a dominant route of exit of the inhibitor from the binding pocket with a coupling to conformational changes in the “flap” of PMX, a β-hairpin located above the binding pocket. In the bound complex, the flap adopts a closed conformation in which it tightly interacts with and covers the inhibitor. On the dominant route observed in our simulations, unbinding involves an open conformation of the flap that allows the inhibitor to exit the binding pocket. After unbinding, the flap adopts an occluded conformation in which the binding site is blocked by a bulky aromatic side chain.

## Graphical abstract

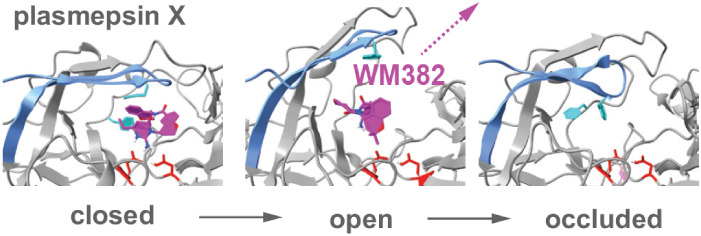

## Introduction

The ten plasmepsins I to X are aspartic proteases that are expressed at different stages of the lifecycle of the malaria-causing parasite *Plasmodium*.^1,2^ In 2017, plasmepsin X was suggested as a promising drug target because of its essential role in the lifecycle.^3,4^ Two potent inhibitors of plasmepsin X are the small molecules WM4 and WM382,^5^ which both contain a guanidinium group. In X-ray crystal structures of PMX in complex with WM382 and WM4, the guanidinium group of the inhibitors faces the two aspartates of plasmepsin X that constitute the catalytic dyad^6^ (see Figure 1). These aspartates are highly conserved in the plasmepsin family and act as a general acid and base in catalyzing the hydrolysis of peptide bonds in substrates.^7,8^ A characteristic structural feature of plasmepsin binding sites is a β-hairpin, called “flap″, that covers substrates and inhibitors in the bound complexes.^9^ In plasmepsin II, the flap adopts an open conformation in X-ray crystal structures of the unbound state, in which the binding site is accessible to ligands.^10^ In plasmepsin X, in contrast, the flap adopts an occluded conformation in crystal structures of the unbound state in which a bulky aromatic side chain of the flap occupies and, thus, blocks the binding site.^6^

**Figure 1:**
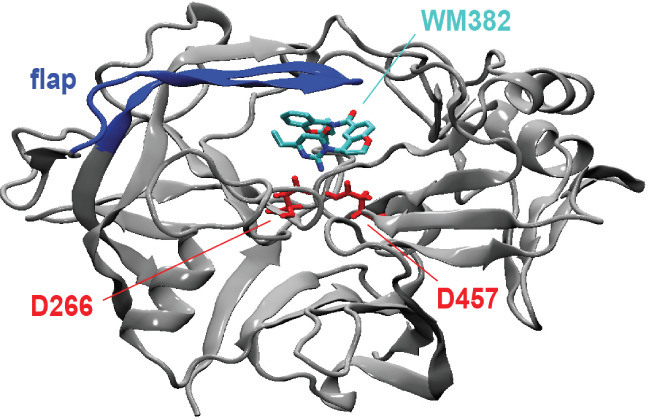
X-ray crystal structure of PMX from *Plasmodium falciparum* bound to WM382.^6^ In this complex, WM382 is in contact with the two catalytic aspartates D266 and D457 and covered by the “flap”, a β-hairpin of PMX. The structure of PMX consists of two typologically similar N- and C-terminal subdomains, which form a crescent shape with a predominant β-sheet core. Each of the subdomains contributes one of the catalytic aspartates. A six-stranded interdomain β-sheet connects the two subdomains.

Here, we first determine the protonation states of the catalytic aspartates of plasmepsin X and the guanidinium group of the inhibitors WM382 and WM4 in the bound complexes. These protonation states are not resolved in the X-ray crystal structures of the complexes due to the lack of hydrogen positions but are required in molecular dynamics (MD) simulations, which we performed to investigate the coupling of the flap dynamics in plasmepsin X to inhibitor binding. For the unbound inhibitors, we obtain p*K*_a_ values of 7.7 and 7.5 for the guanidinium group of WM382 and WM4 with MolGpKa,^11^ a web server for small molecule p*K*_a_ predictions. To identify the shift of these p*K*_a_ values due to the chemical environment of the guanidinium groups in the complexes of WM382 and WM4 with plasmepsin X, we used PROPKA 3,^12^ a widely used standard method for predicting p*K*_a_ shifts of the titratable groups of both proteins and bound ligands based on X-ray crystal structures. For WM382, PROPKA 3 predicts an upward shift of 3.7 of the p*K*_a_ value for the guanidinium group in the bound complex of WM382 and plasmepsin X. The p*K*_a_ value of the WM382 guanidinium group thus shifts from 7.7 to about 11.4 during binding to plasmepsin X, which indicates that the guanidinium group is protonated. The positive charge of the protonated guanidinium group is counterbalanced by the negative charge of the catalytic aspartate D266 predicted by PROPKA 3, while the second catalytic aspartate D457 is predicted to be uncharged in the physiologically relevant pH range. The same protonation states are predicted for WM4 in the complex with plasmepsin X.

To investigate the unbinding pathway of the inhibitor WM382 from plasmepsin X, we performed Hamiltonian replica-exchange MD (H-REMD) simulations. In these simulations, we observed inhibitor unbinding on submicrosecond time scales accessible in atomistic simulations due to the exchanges to Hamiltonian replica energy levels on which the interactions between protein and inhibitor are strongly weakened, rather than on the realistic time scales of seconds or minutes that would be expected for inhibitors with a picomolar inhibition constant such as WM382 (*K*_*i*_ = 38 ± 8 pM).^5^ Along the unbinding pathways in the H-REMD simulations, the flap of plasmepsin X first opens, which allows WM382 to leave the binding pocket. After unbinding, the flap adopts an occluded conformation in the simulations in which it blocks the binding site, in agreement with the crystal structure of unbound plasmepsin X.^6^ The H-REMD unbinding pathways are—strictly speaking—“unphysical” or “alchemical” because they involve unphysical, weakened interactions of the binding partners on Hamiltonian replicas visited along the pathways. However, we propose that the steric requirements suggested for inhibitor unbinding, with the flap opening prior to unbinding, also hold in the physical, realistic system.

## Methods

### System Setup and Relaxation

Because the N- and C-termini of PMX are not resolved in the X-ray crystal structure of the PMX-WM382 complex (PDB ID: 7tbc), we capped the first and last PMX residues of the structure with Ace and Nme residues, respectively, and assigned the protonation states of the titratable protein residues at the physiologically relevant pH of 4.5 with PROPKA 3.^12^ We used the ff14SB force field^13^ for the protein and parametrized WM382 with the General Amber Force Field 2 (GAFF2) and the AM1-BCC method for determining partial charges in the Antechamber package of the Amber20 software suite.^14^ We solvated the protein-inhibitor complex in TIP3P water in a truncated octahedron with the LEaP module of the Amber20 software suite for a 10 Å standard minimum distance between atoms of the protein complex and the edges of the box^14,15^ adding 6 Na^+^ ions to attain charge neutrality of the simulation system. To mimic the physiological salt concentration of 150 mM, we further added appropriate numbers of Na^+^ and Cl^–^ ions for our simulation box size.

To relax the simulation system, we started with a standard two-step energy minimization approach. In the first minimization step, only water and ions were minimized in 5000 steepest descent steps, followed by further 5000 conjugate gradient steps, while the protein–ligand complex was restrained with a harmonic force constant of 100 kcal mol^–1^ Å^–2^ on nonhydrogen atoms. In the second minimization step, all atom positions were minimized in 5000 steepest descent steps, followed by further 5000 conjugate gradient steps. Next, we gradually heated the simulation system from 0 to 300 K in a 500 ps simulation in the NVT ensemble using a Langevin thermostat and harmonic restraints with a force constant of 100 kcal mol^–1^ Å^–2^ on the nonhydrogen atom positions of the protein-inhibitor complex. We further relaxed the system in a 4 ns simulation in the NPT ensemble at 300 K without any positional restraints.

### H-REMD Simulations

We started the H-REMD simulations from the last conformations of five standard-MD trajectories of the complex with a length of 1 μs. Each of these five conformations was used as the initial conformation for four replicas. The H-REMD simulation with 20 replicas had a length of 1 μs and was performed on 20 GPUs in parallel with the pmemd.cuda package of the AMBER20 software suite.^14^ We used the repulsive-scaling H-REMD method introduced by Siebenmorgen, Engelhard, and Zacharias^16^ in which the effective van der Waals radii and the van der Waals attraction in the Lennard-Jones interaction potential *V*_*i*,*j*_ of atoms *i* of the protein and atoms *j* of the inhibitor are modified by the parameters *d* and *ϵ*:

1 $$V_{i,j}^{\prime }=\epsilon \times \epsilon_{i,j}\left[{\left(\frac{R_{min_{i,j}}+d}{r_{i,j}}\right)}^{12}-2{\left(\frac{R_{min_{i,j}}+d}{r_{i,j}}\right)}^{6}\right]$$

In our simulation, we employed the parameter values listed in Table 1, with additional cubic corrections of ϵ_*i*,*j*_ to compensate for effective increases of van der Waals interactions with increasing van der Waals radii.^16^ An isotropic pressure of 1 bar was maintained using a Berendsen barostat with a pressure relaxation time of 2 ps, the temperature was kept at 300 K using a Langevin thermostat with a collision frequency of 1 ps^–1^, bonds containing hydrogen atoms were constrained using the SHAKE algorithm, a cutoff length of 10 Å was used for nonbonded interactions, and long-range electrostatic interactions were calculated with the Particle Mesh Ewald method. In addition, we used hydrogen mass repartitioning^17^ to increase the simulation time step to 4 fs. Exchanges between replicas were attempted at simulation intervals of 0.5 ps.

**Table 1:** Parameters *d* and ϵ of the Rescaled Lennard-Jones Potential in eq 1 for the 20 Replicas of Our H-REMD Simulations

replica	1	2	3	4	5	6	7	8	9	10
*d* [Å]	0.0	0.03	0.06	0.09	0.12	0.15	0.18	0.21	0.24	0.28
ϵ	1	0.99	0.98	0.97	0.96	0.95	0.94	0.93	0.92	0.9

replica	11	12	13	14	15	16	17	18	19	20
*d* [Å]	0.32	0.36	0.4	0.44	0.48	0.52	0.56	0.6	0.65	0.7
ϵ	0.88	0.86	0.84	0.82	0.8	0.78	0.76	0.74	0.72	0.71

### MD Simulations

In addition, we generated five 2-μs-long standard MD trajectories of PMX without a bound inhibitor, starting from the closed PMX structure (PDB ID: 7tbc). The setup and relaxation of this simulation system were conducted with the steps described above for the PMX-WM382 system, and the simulations were performed with the same barostat and thermostat settings, treatment of nonbonded interactions, and simulation time step as the H-REMD simulations.

## Results

### Protonation State Prediction of the PMX-Inhibitor Complexes

In empirical p*K*_a_ predictions of titratable groups of proteins, a general strategy is to predict the shift of the p*K*_a_ values of these groups due to the chemical environment in the protein or protein complex, relative to reference p*K*_a_ values of the isolated groups in solvent. PROPKA 3 is one of the most widely used tools to predict the p*K*_a_ shifts of the titratable groups of both proteins and bound ligands based on X-ray crystal structures or other experimental structures of protein complexes that lack hydrogen positions.^12^ A complication in applying PROPKA 3 to the X-ray crystal structures of plasmepsin X in complex with WM382 and WM4 is that PROPKA 3 only recognizes guanidinium groups as terminal groups, assigning a reference p*K*_a_ value of 11.5 to such terminal guanidinium groups. In WM382 and WM4, however, the guanidinium groups are part of ring structures and not terminal.

To assess the effect of the ring structure in WM382 and WM4 on the p*K*_a_ values of the guanidinium group, we use MolGpKa, a method for predicting p*K*_a_ values of small molecules.^11^ MolGpKa predicts a p*K*_a_ value of 12.7 for the guanidinium group as a single group (see Figure 2), which is somewhat larger than the reference value of 11.5 assigned by PROPKA 3. For a guanidinium group that is covalently bound to a carbonyl group as in the ring structures of WM382 and WM4, however, MolGpKa predicts a strong reduction of the p*K*_a_ value to 6.8. For the complete inhibitor WM382, MolGpKa predicts a p*K*_a_ value of the guanidinium group of 7.7 (see Figure 2). The reduced p*K*_a_ value of the guanidinium group in WM382, compared to guanidinium as a single group, thus appears to result mainly from the carbonyl group that is covalently bound to the guanidinium group. For WM4, MolGpKa predicts a p*K*_a_ value of the guanidinium group of 7.5, which is close to the p*K*_a_ value in WM382 because of the structural similarity (see Figure 2).

**Figure 2:**
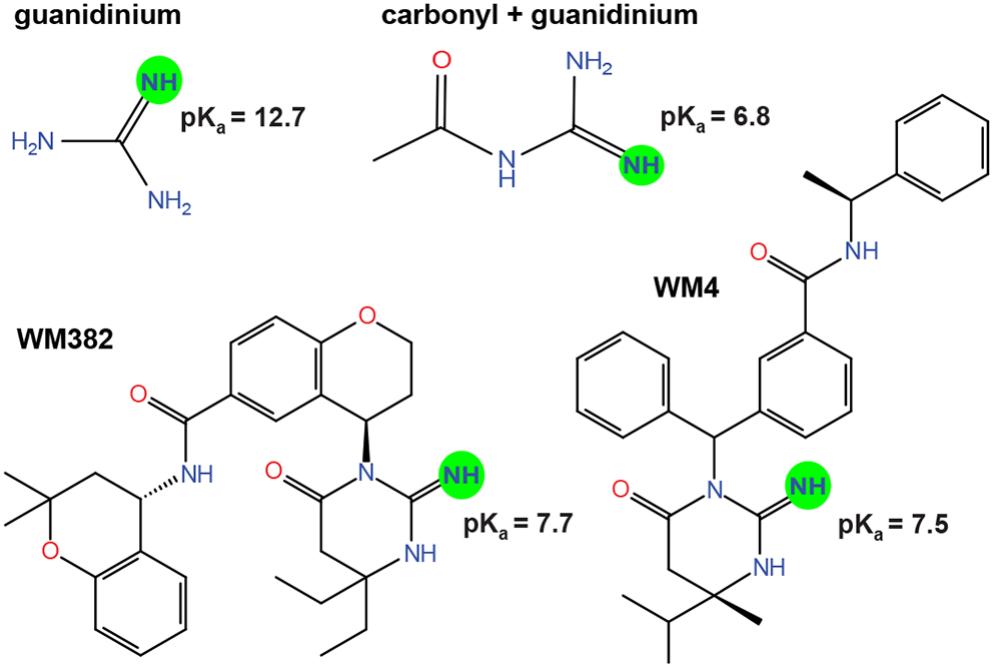
MolGpKa prediction of p*K_a_* of the isolated guanidinium group, the guanidinium group bound to a carbonyl group, and the guanidinium groups of the inhibitors WM382 and WM4.

Because PROPKA 3 does not recognize the guanidinium group in the ring structures of the inhibitors WM382 and WM4, we performed PROPKA 3 calculations with structural segments of the inhibitors. When we retain only the nonhydrogen atoms of the covalently bound guanidinium and carbonyl groups of the inhibitor WM382 in the X-ray crystal structure of plasmepsin X in complex with WM382, PROPKA 3 predicts a p*K*_*a*_ shift of +3.7. The same p*K*_*a*_ shift is predicted by PROPKA 3 when larger fragments of WM382 are retained in the crystal structure of the complex. Taking the MolGpKa-predicted guanidinium p*K*_*a*_ of 7.7 for the unbound inhibitor WM382 (see Figure 2) as the correct reference value, we obtain p*K*_*a*_ ≈ 7.7 + 3.7 ≈ 11.4 for guanidinium of WM382 in complex with plasmepsin X, which indicates that the guanidinium group is protonated. The positive charge of the protonated guanidinium group is counterbalanced by a negative charge of the catalytic aspartate D266 predicted by PROPKA 3, while the second catalytic aspartate D457 is predicted to be uncharged in the physiologically relevant pH range. The same protonation states are predicted for WM4 in complex with plasmepsin X.

### Unbinding Pathways of WM382 in Hamiltonian Replica Exchange MD (H-REMD) Simulations

To investigate the unbinding pathways of WM382 from PMX, we performed an H-REMD simulation starting from the relaxed complex in the protonation state predicted in the previous section (see Methods for details). The p*K*_a_ values of the catalytic aspartates predicted by PROPKA 3 for unbound PMX (PDB ID 7tbb) are 3.9 for D266 and 8.2 for D457 and, thus, are rather close to the predicted p*K*_a_ values in the complex (see Figure 3), which indicates that the dominant protonation states of the catalytic aspartates and of WM382 (see predictions in Figures 2 and 3) do not change in the relevant pH range^2^ from 4.5 to 7 during unbinding. The H-REMD simulation had a length of 1 μs and included 20 Hamiltonian replicas of the system, in which the interactions of plasmepsin X and WM382 are gradually weakened. Exchanges between neighboring replicas were attempted every 0.5 ps and accepted or rejected with a standard exchange criterion that ensures detailed balance. From this H-REMD simulation, we constructed the 20 coordinate-continuous trajectories that are obtained by following the 20 initial conformations along their exchanges between the Hamiltonian replicas. WM382 unbound on 8 of these 20 coordinate-continuous trajectories. Figure 4 illustrates the root-mean-square-deviation of WM382 (RMSD_WM382_) along the 8 unbinding trajectories relative to the WM382 conformation in the crystal structure of the complex, after alignment of PMX. For RMSD values smaller than about 2 to 3 Å, WM382 adopts native-like bound conformations. For RMSD values smaller than about 15 to 20 Å, WM382 is still in contact with PMX in intermediate bound conformations. For RMSD values larger than 20 Å, WM382 is unbound. On 3 of the 8 coordinate-continuous unbinding trajectories (numbered 1 to 3 in Figure 4), WM382 reaches the unbound state from the initial native-like bound state within 15 to 30 ns. On trajectories 4 and 5 in Figure 4, WM382 unbinding occurs at about 80 and 110 ns, respectively. On the remaining trajectories 6 to 8, unbinding takes place between 200 and 400 ns. On several of the unbinding trajectories, WM382 stays in binding intermediate states with RMSD values smaller than 15 to 20 Å for some time prior to unbinding, e.g., on trajectories 5, 6, and 8.

**Figure 3:**
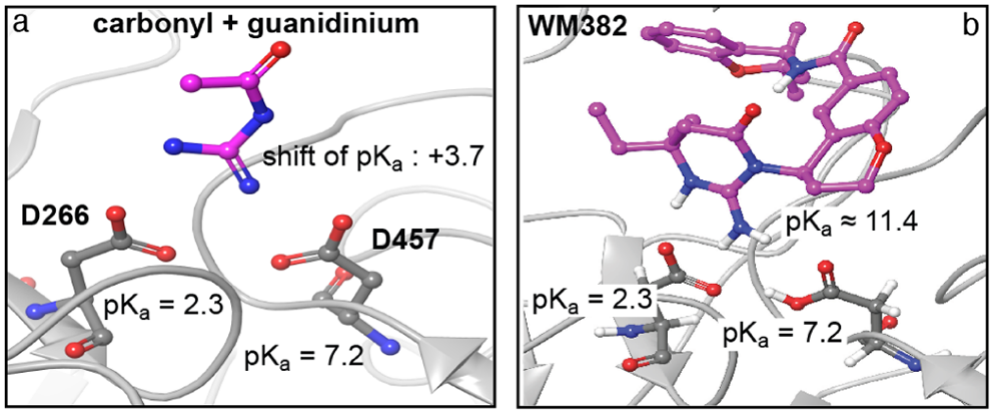
(a) PROPKA 3 ^12^ predicts a p*K_a_* shift of +3.7 for the covalently bound guanidinium and carbonyl groups of the inhibitor WM382 in the X-ray crystal structure of plasmepsin X in complex with WM382. (b) With the p*K_a_* value of 7.7 predicted by MoGpKa^11^ for the guanidinium group of the unbound inhibitor WM382, we obtain p*K_a_* ≈ 7.7 + 3.7 ≈ 11.4 for guanidinium of WM382 in complex with plasmepsin X. Hydrogens of nitrogen atoms in WM382 and at the side chains of the catalytic aspartates D266 and D457 are depicted for the physiologically relevant pH range.

**Figure 4:**
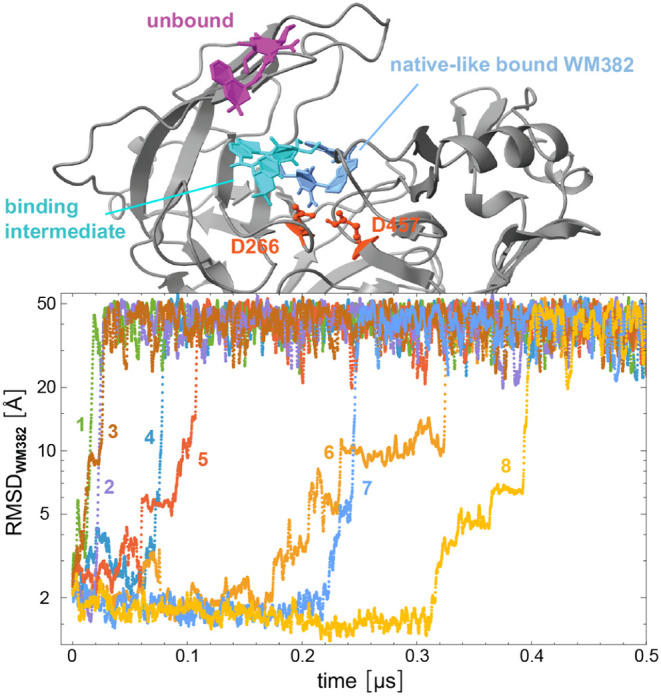
Root mean square deviation of WM382 (RMSD_WM382_) relative to the WM382 non-hydrogen-atom coordinates in the X-ray crystal structure of the complex with plasmepsin X along the 20 coordinate-continuous trajectories of the H-REMD simulation with 20 replicas. On 8 of the 20 trajectories, the ligand unbinds and attains large RMSD values relative to the native binding position. All observed unbinding events occur within the first 0.5 μs of the H-REMD run with a total length of 1 μs. Here, the RMSD_WM382_ values are smoothened by averaging over 11 frames at intervals of 0.1 ns. Representative snapshots of WM382 in the native-like bound state, in an intermediate state, and in the unbound state are shown in blue, cyan, and magenta, respectively.

Along the 8 unbinding trajectories of our H-REMD simulation, WM382 unbinding occurs after an opening of the β-hairpin flap that covers the inhibitor in the native-like bound state. In Figure 5, the distance between the C_α_ backbone atoms of the catalytic aspartate D266 and phenylalanine F311 located at the tip of the flap is plotted versus the RMSD value of WM382 along the 8 unbinding trajectories. At the smallest RMSD values of WM382, the C_α_ distance of D266 and F311 adopts values around 14 Å as in the crystal structure of the PMX-WM382 complex, in which this distance is 13.8 Å. In binding intermediate states with RMSD values of WM382 between about 5 and 20 Å, the C_α_ distance of D266 and F311 attains values up to 20 Å, and on unbinding trajectory 6, even values up to 22 Å, which are clearly larger than the values around 14 Å in the native-like bound state and, thus, indicate an opening movement of the flap. After unbinding, i.e., for RMSD values of WM382 larger than 20 Å in Figure 5, the C_α_ distance of D266 and F311 predominantly adopts values smaller than 14 Å, which indicates a slight movement of F311 into the binding pocket, thus occluding the pocket. This occlusion of the binding pocket observed in our simulations after WM382 unbinding is in agreement with the crystal structure of unbound WM382, in which the C_α_ distance of D266 and F311 is 12.9 Å,^6^ and is accompanied by a reorientation of the terminal benzol ring of the F311 side chain. In Figure 6, the F311 ring orientation along our unbinding trajectories is quantified as the angle between the normal vector of the ring perpendicular to the ring plane and an axis that runs through the C_α_ atoms of D266 and F311. In bound states of WM382 with RMSD values smaller than about 15 Å, the F311 ring orientation angle attains values predominantly around 20°, which indicates that the perpendicular direction of the ring tends to be aligned to the axis connecting the C_α_ atoms of D266 and F311. In the unbound state, in contrast, the F311 ring orientation is distributed over a rather wide range of angles with a distribution maximum at 90° both in the H-REMD simulations and in standard MD simulations of unbound PMX (see Figure 7), which indicates that the F311 ring protrudes into the binding pocket and thus contributes to steric blocking. After inhibitor unbinding, the conformational dynamics of PMX in the H-REMD simulations is not affected by the modifications of the interactions of PMX and WM382 in the different replicas (see Methods). As expected, the distribution of the C_α_ distances between D266 and F311 obtained from the H-REMD simulation trajectories after inhibitor unbinding thus agrees with the distribution obtained from standard MD simulations of unbound PMX within the statistical errors (see Figure 7a). These standard MD simulations were started from the closed crystal structure (PDB ID 7tbc), in which the C_α_ distance of D266 and F311 is 13.8 Å, but quickly adopt occluded conformations with significantly smaller C_α_ distances in the initial 100 ns of the 2 μs-long trajectories. The distribution of the F311 ring orientation obtained from the H-REMD simulations after inhibitor unbinding is somewhat wider than the corresponding distribution obtained from the MD simulations, likely due to sampling limitations within the finite simulation times, which are also reflected by the relatively large standard errors in Figure 7. The F311 ring orientation and the C_α_ distance of D266 and F311 in the unbound, occluded state are rather weakly correlated, with a Pearson correlation coefficient of about −0.25 in both the H-REMD and MD simulations (see Figure 7c).

**Figure 5:**
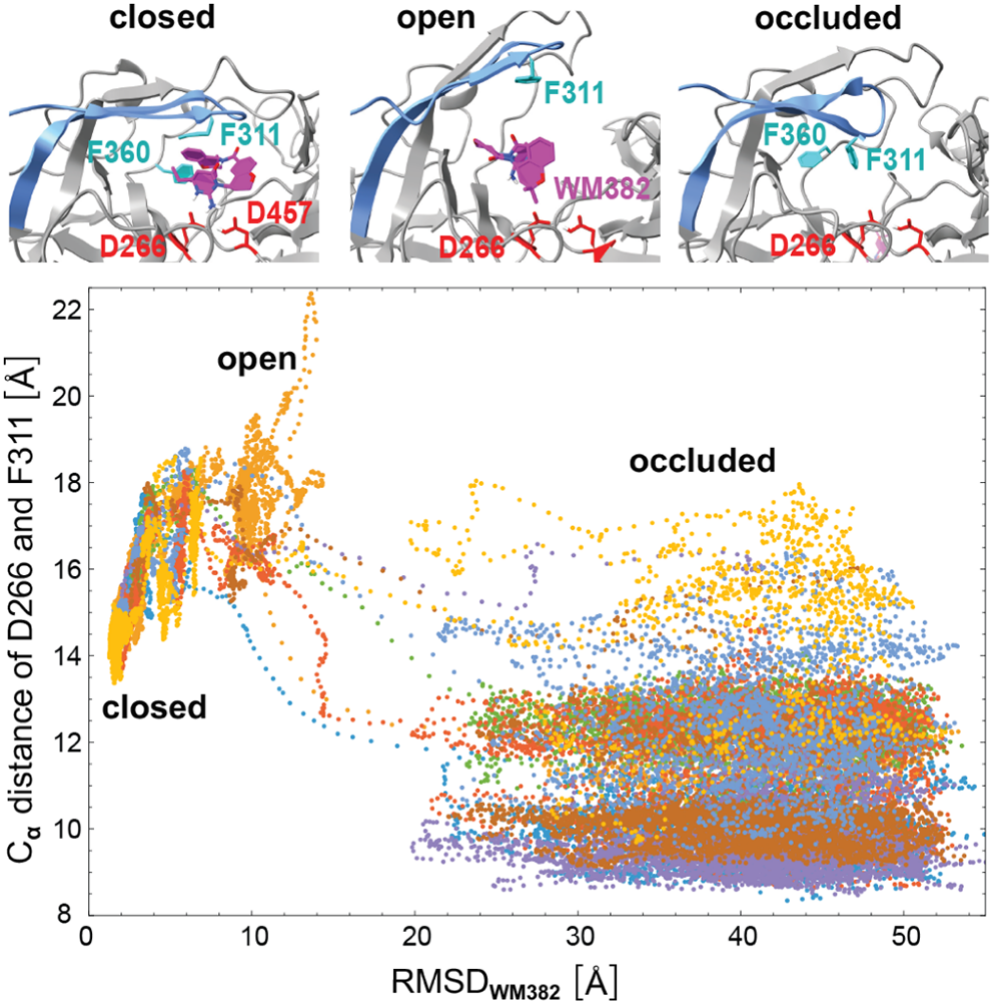
C_α_ distance of the catalytic aspartate D266 and phenylalanine F311 at the tip of the flap versus the RMSD value of WM382 relative to the bound complex along the 8 unbinding trajectories of Figure 4. The C_α_ distances and RMSD values are averages over 11 simulation frames at intervals of 0.1 ns. In the exemplary conformations shown at the top, the C_α_ distance of D266 and F311 is 15.1 Å (closed), 20.5 Å (open), and 11.6 Å (occluded).

**Figure 6:**
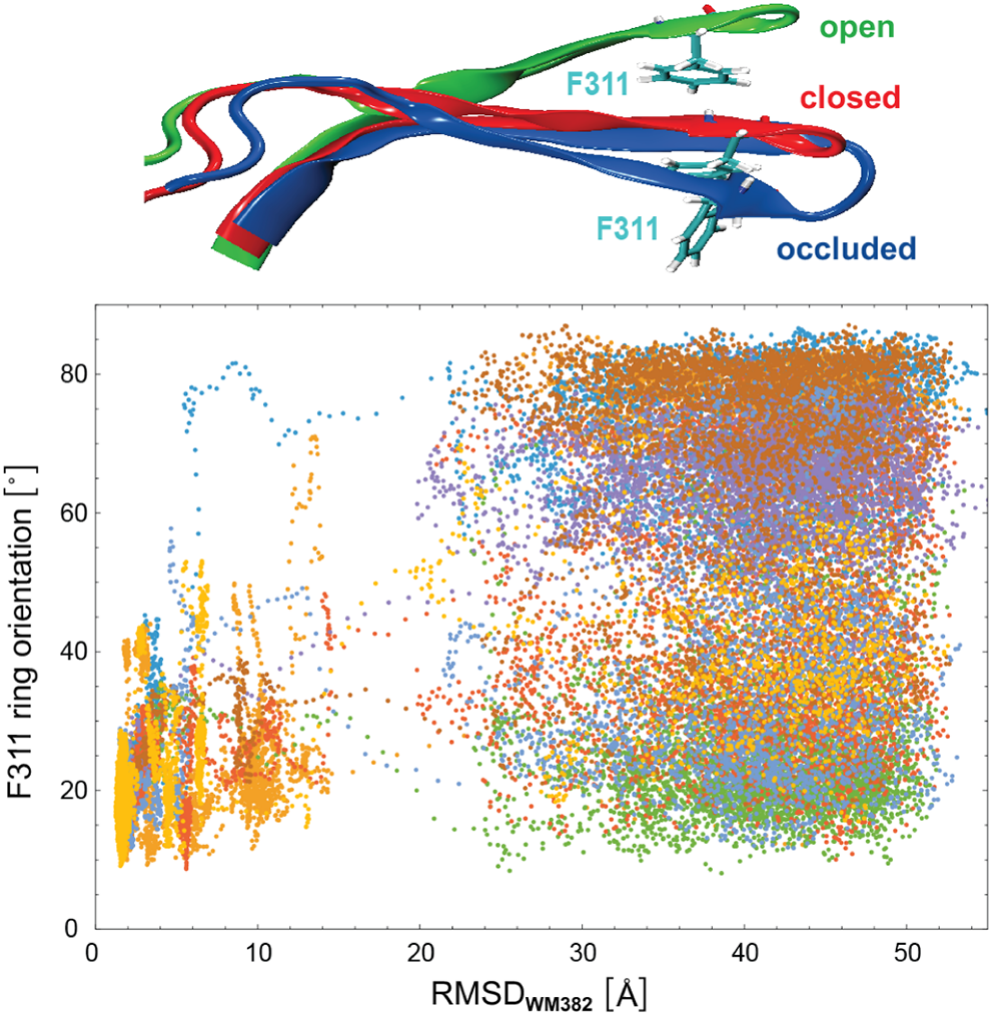
F311 ring orientation angle versus the RMSD value of WM382 relative to the bound complex along the 8 unbinding trajectories of Figure 4. Here, the F311 ring orientation angle is defined as the angle between (a) an axis that is perpendicular to the plane of the terminal benzol ring of phenylalanine F311 and (b) an axis connecting the C_α_ atoms of D266 and F311. The F311 ring orientation angles and RMSD values are averages over 11 simulation frames at intervals of 0.1 ns.

**Figure 7:**
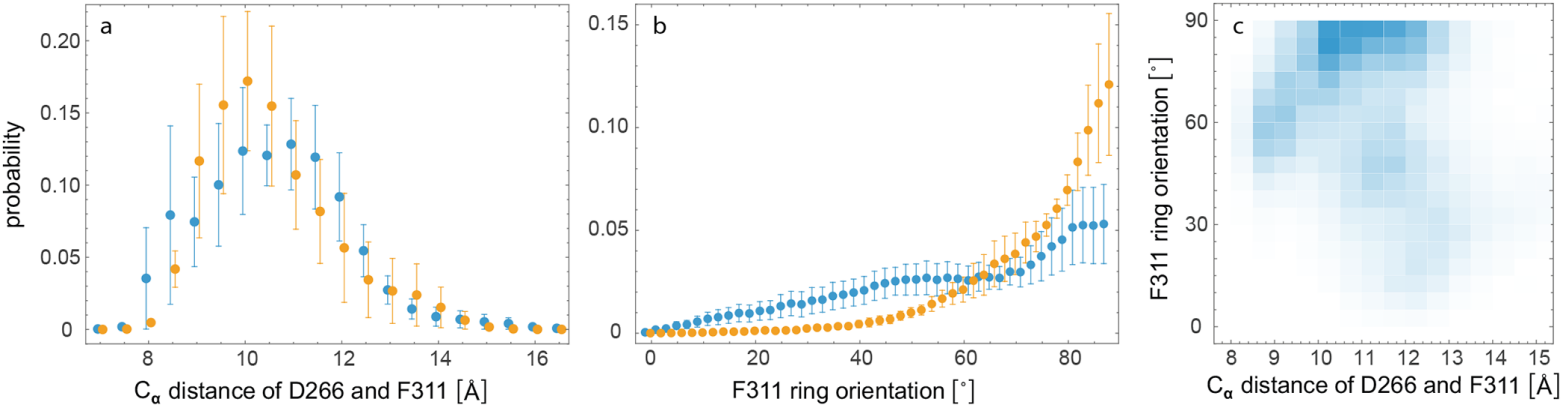
Probability distribution of (a) the distance of the C_α_ atoms of D266 and F311 and (b) the F311 ring orientation angle of Figure 6 in the final 0.5 μs of the 8 H-REMD unbinding trajectories of Figure 4 (blue data points) and, thus, after inhibitor unbinding, as well as in the final 1.5 μs of 5 standard MD simulation trajectories of unbound plasmepsin X with a total length of 2 μs (yellow data points). Here, errors were determined as the standard error of the mean of probability distributions for the individual trajectories. (c) Two-dimensional probability distribution of the C_α_ distance of D266 and F311 versus the F311 ring orientation angle in the final 0.5 μs of the H-REMD unbinding trajectories.

## Discussion and Conclusions

The unbinding pathways observed in our H-REMD simulations can be seen as “unphysical” or “alchemical” because they involve unphysical, weakened interactions of the binding partners on Hamiltonian replicas visited along the coordinate-continuous trajectories. However, it is important to note that the weakening of the interactions between the binding partners rather introduces a bias toward direct unbinding from closed conformations in situations where such direct unbinding is available as an alternative route to unbinding after opening.^18^ Because we do not observe such direct unbinding from the closed-flap conformation along our eight unbinding trajectories for weakened interactions of the binding partners, we conclude that such direct unbinding also does not occur for the original physical interactions of the binding partners. In other words, the reasons for the flap opening prior to inhibitor unbinding observed along our unbinding trajectories appear to be steric, rather than energetic: The exit of the inhibitor appears to be sterically blocked by the closed flap and is only possible if the flap opens, even at the strongly weakened interactions of our H-REMD scheme at which the inhibitor has a strong tendency to unbind.

Because thermodynamic principles imply that the binding pathway of PMX and WM382 is the reverse of the unbinding pathway, our simulation results indicate that WM382 can bind only to PMX conformations with an open flap. Both the closed and occluded conformations of the flap appear to sterically block inhibitor access to the binding site. The conformational flap dynamics of PMX thus also appears to affect the binding rates because binding in the predominantly populated occluded conformation of the flap is not possible. Binding requires an open conformation of the flap, which implies that the binding rates are proportional to the relative population of the minor, open conformation of unbound PMX. The occluded conformation of PMX thus may also be an autoinhibited inactive conformation.^19^ For plasmepsin II, the flap adopts an open conformation in a crystal structure of the unbound protein,^10^ which is also supported by MD simulations in which the flap is observed to remain open.^20,21^ We have focused here on the unbinding of WM382 from PMX in our H-REMD simulations but expect that WM4 also requires flap opening prior to unbinding because of rather similar sizes and bound PMX conformations. The structural details of the bound complexes of PMX and plasmepsin IX (PMIX) with WM382 have also been previously explored in molecular modeling and atomistic simulations.^22,23^

## Data Availability

The molecular dynamics data of this article are available in the Edmond Data Repository at 10.17617/3.CBYPN8.^24^

## ‡

Centre for Targeted Protein Degradation, School of Life Sciences, University of Dundee, 1 James Lindsay Place, DD1 5JJ, Dundee, UK
